# Production of Metabolites as Bacterial Responses to the Marine Environment

**DOI:** 10.3390/md8030705

**Published:** 2010-03-17

**Authors:** Carla C. C. R. de Carvalho, Pedro Fernandes

**Affiliations:** IBB-Institute for Biotechnology and Bioengineering, Centre for Biological and Chemical Engineering, Instituto Superior Técnico, Av. Rovisco Pais, 1049-001 Lisbon, Portugal; E-Mail: pedro.fernandes@ist.utl.pt

**Keywords:** biosurfactants, siderophores, fatty acids, exopolymeric substances, cellular adaptation

## Abstract

Bacteria in marine environments are often under extreme conditions of e.g., pressure, temperature, salinity, and depletion of micronutrients, with survival and proliferation often depending on the ability to produce biologically active compounds. Some marine bacteria produce biosurfactants, which help to transport hydrophobic low water soluble substrates by increasing their bioavailability. However, other functions related to heavy metal binding, quorum sensing and biofilm formation have been described. In the case of metal ions, bacteria developed a strategy involving the release of binding agents to increase their bioavailability. In the particular case of the Fe^3+^ ion, which is almost insoluble in water, bacteria secrete siderophores that form soluble complexes with the ion, allowing the cells to uptake the iron required for cell functioning. Adaptive changes in the lipid composition of marine bacteria have been observed in response to environmental variations in pressure, temperature and salinity. Some fatty acids, including docosahexaenoic and eicosapentaenoic acids, have only been reported in prokaryotes in deep-sea bacteria. Cell membrane permeability can also be adapted to extreme environmental conditions by the production of hopanoids, which are pentacyclic triterpenoids that have a function similar to cholesterol in eukaryotes. Bacteria can also produce molecules that prevent the attachment, growth and/or survival of challenging organisms in competitive environments. The production of these compounds is particularly important in surface attached strains and in those in biofilms. The wide array of compounds produced by marine bacteria as an adaptive response to demanding conditions makes them suitable candidates for screening of compounds with commercially interesting biological functions. Biosurfactants produced by marine bacteria may be helpful to increase mass transfer in different industrial processes and in the bioremediation of hydrocarbon-contaminated sites. Siderophores are necessary e.g., in the treatment of diseases with metal ion imbalance, while antifouling compounds could be used to treat man-made surfaces that are used in marine environments. New classes of antibiotics could efficiently combat bacteria resistant to the existing antibiotics. The present work aims to provide a comprehensive review of the metabolites produced by marine bacteria in order to cope with intrusive environments, and to illustrate how such metabolites can be advantageously used in several relevant areas, from bioremediation to health and pharmaceutical sectors.

## 1. Introduction

Marine bacteria have to adapt to the physical, chemical and biological conditions found in the oceanic environment, which is reflected in their physiology and biochemical properties. The presence of a relatively high concentration of Na^+^ ions in sea water has influenced the requirement for these ions by most marine bacteria [1]. Extreme halophiles can even have optimal growth conditions at 10% (w/v) NaCl [2]. Marine bacteria are also able to live under depleted nutritional conditions and to survive long periods of starvation [3]. Additionally, the vast majority of the marine environment is characterized by low temperatures (below 4 °C) and high pressures (higher than 100 × 10^5^ Pa), which resulted in psychrophilic and barophilic bacteria [4]. There are even bacterial strains with both elevated temperature and alkaline pH optima, being considered poly-extremophiles [2]. Bacterial associations with marine plants and animals have also favored bioluminescent bacteria and in some cases the host organism specifically selects some species of luminous bacteria, preventing the establishment of other species [5].

The diversity of marine environments has exerted a driving force on bacteria selection leading to new adaptive strategies and the synthesis of new metabolites [6,7]. Many of these latter compounds are produced by the bacteria without an apparent function in the growth and development of the bacterial cells and are referred to as secondary metabolites.

Adaptations to the sea conditions can affect secondary metabolite production. For example, of four actinomycetes isolates that produce novel metabolites, all grew to some extent when seawater was replaced by complex fermentation medium in deionized water, but only one of the four was able to produce the metabolite in the absence of seawater [6]. The first bioactive compound produced by a marine actinomycete, an antibiotic obtained from *Chainia purpurogena*, was reported in 1975 [8]. The production of the metabolite was dependent on the addition of an extract of the seaweed *Laminaria*, proving that the production of secondary metabolites by marine bacteria could depend on the presence of nutrients from the marine environment.

The quest for interesting bioactive natural products from marine strains has been stimulated by the need for anti-tumor and anti-cancer agents and for efficient antibiotics for multi-resistant strains. Furthermore, it has been suggested that metabolites obtained from algae and invertebrates such as sponges, mollusks and tunicates are in reality produced by microorganisms [9]. Curiously, the 659 marine bacterial compounds described between 1997 and 2008 were isolated from five bacterial phyla (Bacteroidetes, Firmicutes, Proteobacteria, Cyanobacteria and Actinobacteria), which are the same phyla isolated in parallel studies with terrestrial samples [10].

Molecular approaches such as the extraction of DNA from seawater have shown a much wider bacterial diversity than that obtained by culture-based methods. Cultivation conditions in the laboratory seldom reflect natural conditions found in the sampling site and thereby fast-growing strains are selected (for a review see [11]). The understanding of the mechanisms used by bacteria in the diverse marine microhabitats will allow the use of the proper selection pressure to maintain a high production level of commercially interesting compounds.

## 2. Adaptive Mechanisms

### 2.1. Adaptation at the cellular membrane: Production of specialized lipids

The maintenance of the integrity of the cellular membrane is vital to bacterial cells, since it acts as a permeability barrier to solutes, is responsible for the maintenance of the energy status of the cells, for signal transduction and other energy-transduction processes and for keeping turgor pressure [12]. The ability of bacteria to maintain biological functions under stressful conditions results from changes in protein, sterol, hopanoid and carotenoid content, but mainly from changes in membrane lipid composition [13,14]. The cells try to keep the membrane fluidity by making changes in the degree of saturation of the fatty acids of the membrane phospholipids through a mechanism called “homeoviscous adaptation” [15]. Bacterial adaptation may involve isomerisation of *cis* to *trans* fatty acids, changes in the saturated to unsaturated fatty acid ratio and in the long-chain to short chain fatty acid ratio, modifications in the phospholipid headgroups and in alteration in the outer membrane proteins and lipopolysaccharides [16–18]. In marine environments, in order to survive under extreme temperature, pressure and salinity conditions, bacteria have to be able to adapt their membranes to maintain biological functions. Among the adaptive mechanisms described, those leading to modifications of the membrane to keep the same level of fluidity have been reported most intensively.

In general, higher salinity concentrations cause an increase in the content of negatively charged phospholipids at the expense of neutral phospholipids. Gram-negative bacteria decrease the proportion of zwitterionic phosphatidylethanolamine (PE) in the membrane with simultaneous increase in the proportion of the negatively charged phosphatidylglycerol (PG) and/or diphosphatidylglycerol (cardiolipin, CL) [19,20]. The changes in polar lipid composition could preserve the membrane bilayer structure, since PG forms bilayers whilst PE containing unsaturated fatty acids tends to form non-bilayer phases. In Gram-positive bacteria, the anionic lipid fraction increases with salinity due to an increase in the percentage of CL rather than of PG [20]. Changes in the cellular membrane that confer an advantage under a certain stress may also further help the cells. For example, strains that have membranes with a high degree of saturation because they were grown under high pressure conditions will be better adapted to compete for available nutrients under high salinity conditions.

The outer layer of Gram-negative bacteria is mainly constituted of lipopolysaccharides (LPS), which are usually organized into three subdomains: a glycolipid region (lipid A), the oligosaccharide of the core, and a polysaccharide referred as *O*-specific polysaccharide (OPS). The OPS of marine bacteria are often anionic in what could be seen as an adaptation to the marine environment: negative sites on the polysaccharide allow the formation of ionic interactions mediated by divalent cations [21]. This results in a stronger membrane packaging, confering an increased stability towards environmental stress such as high pressure. The shortening of the fatty acids in lipid A has been observed as a short term response to cold stress in mesophilic bacteria and the shortening of fatty acids in the glycolipids could be seen as an evolutionary adaptation of obliged psycrophiles [21].

Bacteria are also masters at changing the fatty acid composition of their membranes in response to environmental stresses. *Oceanimonas baumannii* presented two different adaptive stages with increasing salinity: at 0.15–1% NaCl (w/v) the ratio of zwitterionic:anionic phospholipids increased and the percentage of saturated fatty acids decreased; whereas at 1–7% NaCl the zwitterionic:anionic phospholipids ratio decreased but the saturated:unsaturated fatty acid ratio was unaffected [22]. An increase in the saturation degree of the membrane phospholipids of bacteria growing in high concentrations of salt will decrease the fluidity of the cell membrane and will make the cell more resistant to bursting under osmotic shock [23].

Oil spills occur often in ecosystems with high or moderate salinities, such as the sea, estuaries and seashores, and thus bioremediation of alkanes and other oil compounds must be carried out under saline and non-optimal temperature conditions. Since both salinity and growth temperature influence the cellular hydrophobicity, the uptake of the hydrocarbons can also be quite influenced [24] and thus the rate at which a site is cleaned.

In phsychrophiles, the very cold rigidifying conditions are overcome by incorporation of specific fatty acids to maintain membrane fluidity and transport of substrates and nutrients across the membrane. In the Gram-positive *Marinococcus halophilus*, an increase in salinity or temperature had the same response: an increase in the content of saturated fatty acids (mainly 18:0) and a decrease in the content of the branched chain 15:0 ante-iso fatty acids [20].

Bacteria have traditionally been considered unable to produce poly-unsaturated fatty acids (PUFAs), but deep sea isolates have been found to contain PUFAs such as docosahexaenoic acid (DHA) and eicosapentaenoic acid (EPA) in their membranes. Bacteria containing PUFAs have been isolated from deep sea fish, sediments, seawater and amphipods [25]. The response of a facultatively barophilic strain to increased pressure and also to low temperature included a decrease in the amount of saturated fatty acids in PE and an increase in PUFAs, including DHA [25]. In PG, the decrease in saturated fatty acids was accompanied by an increase in DHA. Similar patterns were observed in an obligately barophilic strain, suggesting that unsaturated fatty acids including DHA are important in maintaining fluidity conditions under high pressure. However, other studies have shown that the role of DHA and EPA are not exclusive in adaptation to high pressure, and could also influence adaptation to temperature and salinity. Cells of *Vibrio marinus* MP-1 grown at 20 °C contained only 17% of the DHA produced when the cells were grown at 5 °C [4]. However, the work of Valentine and Valentine suggested that the cells are able to choose from a wide array of phospholipids structures to maintain membrane fluidity in different environments [26]. Omega-3 bacteria have at least three distinct routes for producing fluidizing lipids such as methyl-branched and mono-unsaturated lipids in addition to the EPA and DHA routes. Since pressure, temperature and the concentration of ions affect a variety of biological functions, biological adaptation should not be a single-site phenomenon.

Bacterial cells are also able to produce fatty acids as defense compounds. For example, *Pseudoalteromonas haloplanktis* produces isovaleric acid (3-methylbutanoic acid) and 2-methylbutyric acid (2-methylbutanoic acid), which have antibacterial activities [27].

**Figure 1:**
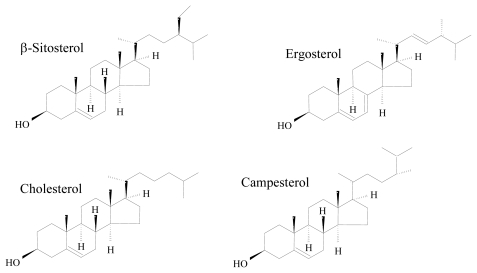
Chemical structure of common sterols.

Sterols play a relevant role in the physiology and biochemistry of all eukaryotic organisms. As part of the cell membrane, they control its fluidity and permeability. Besides this well-established role, sterols can be involved in the control of membrane related metabolic processes, are the precursors for a wide array of relevant metabolites for cellular and developmental processes (*i.e.*, steroid hormones, ecdysteroids), and have also been reported to be involved in signal transduction events in higher organisms [28–30]. Examples of the structure of some common sterols are given in Figure 1. Marine monohydroxy sterols present however a wider diversity than those from terrestrial sources, given the large number of substitutions and rearrangements, which lead to a vast array of side chains [29,30]. Some marine microorganisms, namely microalgae, were shown to change the sterol concentrations as response to environmental stimuli [31].

With the notable exception of members of the family *Methylococcaceae*, bacteria do not produce sterols, but they do produce hopanoids: pentacyclic compounds that play a role similar to sterols at the cell wall level [32,33]. Thus, hopanoids have been suggested to contribute to ruling membrane fluidity and to decreasing the diffusion of ions across the cell membrane [34]. The fact that the hopanoid content of some bacteria increases in response to environmental stress, such as to temperature increases and to the presence of alcohols, seems to support such claims [35,36]. Recently, however, some controversy has arisen, given the relatively low number of potential hopanoid-producing bacteria, which suggests that hopanoids may play a yet unidentified key role in bacteria [37]. Hopanoids have typically been regarded as derivatives of aerobic organisms. Nevertheless, their biosynthesis has been observed in several anaerobic bacteria [38], one of these being *Desulfovibrio bastinii*, a sulfate-reducing bacteria widely found in marine environments [39].

### 2.2. Production of exopolymers and biosurfactants

Bacterial exopolysaccharides, or more generally exopolymeric substances (EPS), have several functions including protection from desiccation, cryoprotection, stabilization of enzymes by buffering pH and salinity fluctuations, surface adherent, nutrient storage and sequestration of toxic compounds. Polysaccharides represent in general 40–95% of the EPS [40], but proteins, nucleic acids and lipids can also be found in the matrix formed by cells in biofilms and aggregates. The majority of the marine bacteria studied produce heteropolysaccharides with three or four different monosaccharides (e.g., pentoses, hexoses, amino sugars or uronic acids) arranged in groups of ten or less repeating units [41]. Functional groups such as sulfate and phosphate, which are negatively charged above pH 7, may also be present. Negatively charged EPS may bind selectively to dissolved iron (Fe^2+^ and Fe^3+^) acting as an organic ligand [41], decreasing the rate of oxidation of dissolved iron and increasing the bioavailability of iron in the sea water. A new exopolymer from *Pseudoalteromonas* sp. strain TG12 exhibits both high emulsifying activities and the capacity to desorb various mono-, di-, and trivalent metal species from marine sediments, probably due to its high content of uronic acids [42].

The benefit given by the EPS must compensate the important carbon and energy investment required by the bacterial cells for their production [43]. Most of the marine bacteria studied produce the largest quantity of EPS during stationary phase [44], under nutrient (e.g., nitrogen, phosphorous, sulfur, potassium) depletion conditions [45] or in response to environmental stress [41]. The *Pseudoalteromonas tunicata*, which usually forms biofilms on the surface of eukaryotic organisms produces several extracellular inhibitory compounds, including an antibacterial protein alleged to help this bacterium compete for space and nutrients on surfaces [46]. The protection given by the EPS matrix to cells in biofilms will be further discussed in the next section.

Novel EPS have been isolated from marine bacteria, especially from those collected in the deep-sea near hydrothermal vents. This environment is characterized by high pressure and temperature, as well as high levels of sulfur and heavy metals. Several of the mesophilic, thermophilic and hyperthermophilic bacterial strains isolated have been able to produce EPS in the laboratory [47]. When screened for therapeutical use, these EPS have been found useful in tissue regeneration, and for the treatment of cardiovascular and oncological diseases [46]. Some of the properties conferred by the EPS to the cells that produce them and the properties of the EPS that have been exploited are listed in Table 1.

The bacterium *V. diabolicus* produces an unusual EPS with a high percentage of osamines and containing glucuronic acid, N-acetyl-glucosamine and N-acetylgalactosamine [51]. This polymer was found to be a strong bone-healing material in an experimental model. The structure and chemical characteristics of bacterial EPS may also be modified to obtain desired properties. An EPS obtained from *A. infernus* was modified to obtain new heparin-like compounds [51]. The resulting EPS, contrary to the native one, presented anticoagulant properties similar to heparin, suggesting that by carrying out modifications in natural EPS their functionality and specificity may be improved.

Some of the EPS from marine bacteria have biosurfactant properties, decreasing the surface tension of the media and increasing the bioavailability of low water soluble compounds. One of the fractions obtained from the crude biosurfactants of *B. circulans* reduced efficiently the surface tension of water from 72 to 28 mN/m [52]. Additionally, the lipopeptide biosurfactant fraction also presented antimicrobial activity against several Gram-positive and Gram-negative pathogenic and semi-pathogenic strains including some multidrug-resistant pathogenic clinic isolates. The *Halomonadaceae* sp. strain MM1 produces a novel surface-active glycolipid which increases chlorinated biphenyl degradation probably by helping solubilization of these usually low soluble compounds in water [48]. The marine α-proteobacterium *Antarctobacter* sp. TG22 produced an extracellular emulsifying agent, which was found to be a high molecular weight glycoprotein with high uronic acid content [55]. The emulsion-stabilizing properties of the polymer were comparable to that of xanthan gum and gum Arabic for a range of different food oils. *H. eurihalina* F2-7 produces a potent emulsifying agent with pseudoplastic behavior that forms gels at high viscosity at acidic pH [56]. *Halomonas* species produce emulsifiers effective in a wide range of food oils under both neutral and acidic pH conditions, even under high temperature and acidic conditions [57]. More than 200 bacterial strains isolated from oil wells and related environments were able to produce EPS [49]. The predominant facultative anaerobe belonging to the *Bacillus* species, and growing at up to 12% NaCl, produced a polysaccharide (containing glucose, arabinose, mannose, ribose and low contents of allose and glucosamine) with pseudoplastic behavior, and resistance to shear stress and thermal degradation.

### 2.3. Biofilm formation and quorum sensing

As mentioned in the previous section, extracellular polymers enhance the adaptability and survival rate of bacteria in marine environments, especially under extreme conditions. The EPS change the physical and biogeochemical microenvironment around the cell [58]. The matrix of a biofilm can also bind and concentrate metal ions, metalloids and molecules, with the binding and release of metal ions (e.g., Cd^2+^, Cu^2+^, Pb^2+^, Fe^2+^) depending on the EPS composition and physical gel state, on the pH and salinity [59]. Marine biofilm populations can even combat protozoan predators using chemically mediated resistance [60]. Nanomolar concentrations of violacein, an alkaloid produced by biofilm cells, induce a conserved eukaryotic cell death process thus inhibiting protozoan feeding. This mechanism of protection could explain both the persistence of bacterial biofilms in various environments and the production of eukaryote-targeting bacterial metabolites. One key factor for the environmental survival and transmission of *Vibrio* species is the ability to form biofilms [61].

The development of biofilms is thought to be mediated by chemical compounds that act as signals and that are released by bacteria in a density-dependent manner. The process called “quorum sensing” [62] induce synchronized physiological changes in cells by releasing of signals, or auto-inducers, when a threshold concentration is reached. At least five types of quorum sensing (QS) systems have been identified: three “archetypal” systems to Gram-positive and Gram-negative bacteria; a fourth that has been described only in *Myxococcus xanthus*; and a fifth system described in *Pseudomonas aeruginosa* [63]. Since horizontal gene transfer (HGT) is promoted at high bacterial cell concentration and *Roseobacter* species produce acyl homoserine lactones, which are known to be involved in QS in Gram-negative bacteria, Wilson and Salyers questioned if HGT could be enhanced in high cell communities such as biofilms and marine snow [64].

The discovery of *N*-acylhomoserine lactone as a QS signal was first made in the luminescent marine bacteria *V. fischeri* and *V. harveyi*. The LuxI/R system of *V. fischeri* is the paradigm of Gram-negative quorum sensing systems even though it is not found in all vibrios [65]. This could demonstrate the flexibility of the *Vibrio* genome in responding to a myriad of activities under highly variable marine conditions and the role of QS signaling in biological diversification.

The extraordinary diversity of marine metabolites has also showed that some strains can produce inhibitors of QS of other bacteria. *Halobacillus salinus*, a Gram-positive bacterium, secretes two phenetrylamide metabolites able of quenching quorum sensing-controlled processes in several Gram-negative strains including bioluminescence in *V. harveyi*, violacein biosynthesis in *Chromobacterium violaceum* CV026 and green fluorescent protein production in *Escherichia coli* JB525 [66]. Apparently, the two non toxic metabolites compete with N-acyl homoserine lactones for the receptor binding site. The development of quorum sensing inhibitors could help the development of sound strategies against biofilm-forming pathogens.

## 3. Production of Secondary Metabolites

### 3.1. Terpenes and terpenoids

Terpenes, which are compounds derived from isoprene units, are widely spread throughout nature, mainly in plants as constituents of essential oils. For his contribution to the identification and characterization of terpenes from essential oils, Geheimrat Otto Wallach was awarded with the Nobel Prize in Chemistry in 1910. More than 25,000 individual terpenes and terpenoids, their oxygenated derivatives, are known, which makes this probably the largest group of natural products. The large majority of known terpenes are derived from terrestrial sources, in particular from plants and fungi. Studies describing the biotransformation using enzymes, cell extracts or whole cells of bacteria, cyanobacteria, yeasts and microalgae have also been published (for a review see [67]). However, marine terpenes have been scarcely studied, and the knowledge of the biochemical processes involved in their synthesis is still limited except for a few algae and marine invertebrates. Terpenes have a variety of roles in mediating antagonistic and beneficial interactions among organisms and physiological functions (for a review see [68], including membrane stabilization, anti-oxidant properties, signaling and protection.

The majority of the bacteria occurring in the sea are chromogenic. The carotenoids produced by marine bacteria do not differ significantly from terrestrial bacteria, the main characteristics being a comparative lack of hydrocarbon carotenoids, synthesis of polydroxy compounds and inability to synthesize lutein [69]. A gene encoding for lycopene β-monocyclase, which metabolizes the carotene lycopene to γ-carotene, was isolated from strain P99-3 (previously *Flavobacterium* sp.) that produced myxol, a γ-carotene derivative. To understand the variability of proteorhodopsin proteins, which are bacterial retinal-binding membrane pigments that function as light-driven proton pumps in the marine ecosystem, Sabehi and co-workers surveyed large bacterial artificial chromosome libraries from the Mediterranean and Red Seas using Southern hybridization and newly designed general degenerated PR primers [70]. The study showed that bacteria producing proteorhodopsin proteins are an important component of the microbial communities in the photic zone of both seas and that several phylogenetically diverse genes encode functional light-driven proton pumps. These bacteria are well adapted to oligotrophic marine surface waters by exploiting both light and a few reduced organic sulfur compounds for energy production.

**Figure 2:**
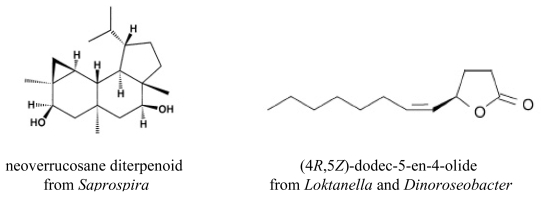
Examples of terpenes produced by marine bacteria.

*Saprospira grandis*, which besides being heterotrophic on organic substrates has the ability to prey on other bacteria that are captured by secreted mucilage [71], produces a series of neoverrucosane diterpenoids (example given in Figure 2), rarely produced by prokaryotes [72,73]. The only bacterium reported to produce diterpenoids, apart from filamentous actinomycetes, is the hyperthermophile Gram-negative bacterium *Chloroflexus aurantiacus* [74]. In this case, the verrucosane-2β-ol produced has apparently a function in modulating membrane fluidity similar to that of hopanoids and steroids in other microorganisms. The major pigment produced by *S. grandis* was identified as the red xanthophyll saproxanthin.

In 2007, Schulz and Dickschat published an extensive review on the production of volatiles by bacteria [75]. Among the myriad of compounds presented, several are produced as secondary metabolites of marine bacteria, including the following: (4*R*,5*Z*)-dodec-5-en-4-olide by the α-proteobacteria *Loktanella* and *Dinoroseobacter* (Figure 2); iso- and anteiso-branched γ- and δ-lactones by *Streptomyces caviscabies*; 2-phenylethanol produced by a marine Artic bacterium, by two *Roseobacter* strains, by *Staphylococcus xylosus*, and several streptomycetes.

The increasing number of terpenes reported from marine bacteria suggests that there should be a high number of these compounds still to be isolated and characterized. Their role in bacterial cell physiology is also unknown in most cases.

### 3.2. Production of siderophores

Most microorganisms depend on iron for diverse cell processes, namely respiration, photosynthesis or production of pigments. In the presence of low-iron environments, such as the ocean seawater, where the dissolved iron level is roughly of 20 pM to 1 nM [76–78], several microorganisms respond by synthesizing low molecular weight molecules (0.4–1.5 kDa) with strong iron binding capabilities. These molecules, termed siderophores, scavenge, solubilize and ultimately transport iron (III) inside the cell [79–81]. Still, it has been suggested that the contribution of siderophores for iron complexation is far more relevant in coastal seawater than in the open ocean, where they only contribute 0.2–4.6% of the dissolved iron complexation capacity [82].

The Fe(III)-siderophore complexes are quite stable, since the stability constants range from 10^25^ to 10^50^ for such metal-ligand complexes [83–85]. This extreme ability of siderophores for iron chelation creates a driving force for its application in food, feed and health. Siderophores are thus being used both for iron nutrition in several organisms [86–92], as well as for reduction in iron overload under given medical condition through deferration [92–96]. The application of siderophores has moved beyond iron overload conditions [97], since iron deprivation is a suitable approach for therapy of breast cancer [98], malaria [99], and microbial infection through immunomodulatory action [10] or through the synthesis of siderophore-antibiotic conjugates (*i.e.*, pyoverdin/β-lactams). In the latter approach, the cell mechanisms for iron uptake are used, so that the iron complex is transported into the cell, allowing the large substituent to decrease the hydrolytic activity of enzymes towards the antibiotic [100]. Promising results were also obtained when siderophores were used for the therapy of other non-iron overload conditions, such as rheumatoid arthritis, cardiac poisoning, solid tumors, hematological malignancies and aluminium related pathologies [92,101]. Siderophores have been furthermore shown to play a role in the solubilization of iron (hydro)oxides [80]. Siderophore production is regulated by quorum sensing in given bacteria [102], but it has also been shown that siderophores can also have some cell signaling function, in particular when coupled to homoserine lactone compounds, already known to be chemical signals for several gram-negative strains [103,104]. Siderophores can thus contribute to intra/interspecies communication in natural seawater. The growth of bacteria harboring no detectable siderophore or homoserine lactone production capability was stimulated by some siderophores (*i.e.*, desferrioxamine, *N*,*N*′-bis (2,3-dihydroxybenzoyl)-*O*-serylserine) produced by other microorganisms in seawater [103,104]. Occasionally the production of siderophores by non-producing strains was induced by siderophores produced by a different species of marine bacteria [103,104]. The enhanced cell growth promoted by the combined use of siderophores and homoserine lactones eased culture isolation, suggesting this approach is prone to detect more diversity by pushing forward the capability for isolation and growth of marine microorganisms *in vivo* [103,104]. Pyoverdine was also shown to act as a signaling molecule by triggering the expression of genes that form part of the regulon in *Pseudomonas aeruginosa*, thus controlling the production of some virulence factors that are excreted by this bacteria [105].

Desferrioxamine B was also shown to form stable complexes with uranium (VI). The different species of the uranyldihydroxo complex formed had stability constants within 10^17^–10^23^ [106]. Some siderophores can also form complexes with boron. It has been thus evidenced that borate and Fe^3+^ actually compete for binding to microbial siderophores [107,108]. Systematic analysis of data gathered on this matter suggests that the relevant functionality for boron binding is the presence of vicinal dianionic oxygen in the binding group (*i.e.*, catecholate or citrate) present in the backbone of the siderophore molecule [109]. Unlike iron, boron is available in relatively high concentrations in marine environments, therefore despite being an essential micronutrient for known dietary requirement for various phytoplankton, boron scarcity is not an issue when the primary growth requirements of marine (micro)organisms is considered. Given the potential toxicity of boron at high concentrations, there is rather the need for control mechanisms in marine species [109]. On the other hand, it has been suggested that the boron binding capability may be required for the resulting siderophore complex to act as a signaling/quorum sensing molecule [107,109]. Boron is also an essential micronutrient for all higher terrestrial plants among other terrestrial organisms. Albeit, boron is again toxic at high environmental concentration, the terrestrial environment is boron-poor, thus boron binding siderophores may be of use in this matter [109]. Siderophores have also been shown to recognize, bind and promote biofilm formation through cell adhesion to metal oxides, such as titanium oxide [110].

The number of siderophore structures from marine microorganisms is relatively scarce when compared with those from terrestrial microorganisms. This may be partially ascribed to the tapping of marine resources for this class of compounds being undertaken more recently. Unlike terrestrially produced siderophores, those originated from marine microorganisms are vastly amphiphilic [111,112]. It has been suggested that this latter feature allows the binding of siderophores to the cell membrane, preventing its loss by dilution in the marine environment [111]. Siderophores naturally present in seawater have been isolated from sampling sites in the Atlantic Ocean. Ferrioxamines G and E, particularly the former, were widely distributed in the sites in dissolved form. Amphibactins were also identified in the supernatant of cultures of Atlantic Ocean seawaters incubated under controlled conditions, during the stationary phase. Amphibactins were not detected in dissolved form otherwise. Again this overall behavior can be ascribed to the particular nature of the siderophores, since ferrioxamines have a hydrophilic nature, and amphibactins are associated to cell membrane, given their amphiphilic nature [113]. On the other hand, ferrioxamines, which are hydroxamate siderophores, are the most stable marine siderophores and do not react when exposed to sunlight, regardless of Fe(III) complexation [114]. Ferrrioxamines and other non-photoactive siderophores can thus contribute significantly for increasing the residence time of iron in surface water as well as to induce the dissolution of iron-containing materials. The ability of siderophores produced by the marine bacterium *Alteromonas haloplanktis* to dissolve ferric hydroxides illustrates the latter concept [115]. Borer and co-workers [80] also showed that even in the absence of electron donors, aerobactin accelerated the light-induced dissolution of Fe(III) oxides, an effect that was ascribed to the efficient transfer of reduced surface Fe(II) into solution, alongside with the photolysis of the corresponding surface complexes. These findings clearly establish siderophores as one of the players in the dissolution of iron-containing materials in the marine systems.

Two major patterns for the wide majority of marine sidephores have been pinpointed. On the one hand, are suites of amphiphilic siderophores containing a Fe(III)-binding headgroup and a fatty acid appendage with variable chain length. On the other hand, are siderophores containing α-hydroxycarboxylic acid moieties (*i.e.*, citric acid or β-hydroxyaspartic acid), which are photoreactive when coordinated to Fe(III) [116,117]. Most of the siderophores with known structures are produced by α- and γ-proteobacteria (*i.e.*, *Halomonas* spp., *Marinobacter* spp., *Vibrio* spp.) [112,118]. Cyanobacteria have not typically been known to produce many siderophores, and only a limited number of these compounds from coastal cyanobacteria has been characterized [116,119–124]. Among these are anachelin, schizokinen and synechobactin [124], that have been furthermore suggested as promising metabolites for the development of therapeutic drugs for the treatment of persistent metal toxicity [124]. There are several heterotrophic bacteria and fungi that also produce siderophores [125–127]. Within the photosynthetic picocyanobacteria and eukaryotic microorganisms that inhabit the open ocean no siderophore production has been detected, suggesting that these organisms adapted to the diluted ocean environment by developing uptake pathways involving a reduction step to free bound iron [124,128]. Whereas the full identification of siderophores from bacterial sources is well established, the full identification of siderophores from marine fungi is lagging behind. Thus, although 24 marine fungal strains have been shown to produce siderophores, only those from *Penicillium bilaii*, from *Cuninghamella elegans* ATCC36112 and from *Aureobasidium pullulans* HN6.2 are well characterized [127,129]. Examples of all these are given in Table 2.

When bacteria are under iron starvation, siderophores bind to Fe^3+^, yielding a thermodynamically very stable ferrisiderophore, [146]. The resulting complex transports iron inside the cell, across the outer and inner membrane, in an energy-dependent process, where ATP hydrolysis takes place [81,112]. For this uptake to occur, outer membrane receptor proteins are required to selectively recognize the complex formed [147]. Once the intracellular concentration of iron matches the titer required to cope with the physiological demand, the biosynthesis of both siderophores and receptor protein are repressed [139]. Extensive information on the mechanisms of iron uptake by microorganisms can be found elsewhere [148]. Catecholates, hydroxamates, and α-hydroxycarboxylates and, to a lesser extent, carboxylate groups, are the most common binding groups of bacterial siderophores [80]. Typical coordination mechanisms of Fe(III) with marine siderophores are given in Table 3.

As part of natural iron chelators, marine siderophores are clearly relevant players in aquatic ecosystems due to the role played in the iron cycle. They can furthermore contribute to the development of therapeutic drugs and food supplements, targeted at specific conditions, as well as in the development of biosensors and biofilms [149].

## 4. Final Remarks

The full chemical potential of marine bacteria is still far from established. Recent technological improvements have considerably enhanced this field of studies, for example, high pressure liquid chromatography with diode array detection for metabolite assessment, mass spectrometry and nuclear magnetic resonance to determine the identity and structure of the compounds and even the possibility of analyzing whole-cell MALDI-TOF MS spectral profiles. The use of fluorescent specific dyes to assess cellular physiology and the structure of cell aggregates and biofilms will also contribute to the study of the particularities of adaptive mechanisms in marine bacteria.

The major obstacle to the full assessment of bacterial metabolites and adaptive mechanisms is, however, the difficulty to reproduce in laboratory the conditions usually found by the bacteria in natural stressful environments. Unfortunately, it is still common to find differences in bacterial diversity measured from the counting of cultures obtained in the laboratory from natural samples and the diversity assessed directly in those samples by the application of molecular techniques. Hopefully, the recent advances in analytical methodologies will enable us to clarify the particular biochemical mechanisms and potential of marine bacteria.

## Figures and Tables

**Table 1:** Functions and applications of exopolymeric substances produced by marine bacteria.

Examples of functions of EPS in bacterial cells			
*Type of EPS*	*Function*	*Bacterium*	*Reference*
Glycolipid	Biosurfactant	*Halomonadaceae* sp. strain MM1	[48]
Polysaccharide	Benefit during competition for space and nutrients on surfaces	*Pseudoalteromonas tunicata*	[46]
Polysaccharide	Allow survival in oilwells	*Bacillus* sp.	[49]
Polysaccharide and proteins	Helps microbial interactions	*Nocardia amarae*	[50]

**Examples of applications of EPS from marine bacteria**			
*Bacterium*	*Applications*		*Reference*

*Alteromonas infernus*	Bone-healing material		[51]
*Bacillus circulans*	Biosurfactant; antimicrobial action		[52]
*Vibrio* and *Alteromonas*	Tissue regeneration; antithrombotic effects		[46]
*P. tunicata*	Antifouling activity		[53]
*Flavobacterium uliginosa*	Antitumor activity		[54]
*Bacillus* sp.	Pseudoplastic behavior		[49]

**Table 2:** Some siderophores produced by marine microorganisms that have been recently described. Apart from their common Fe(III) scavenging nature, other (potential) roles or noticeable features are referred to.

Siderophore	Producer	Comments	Reference
Aerobactin	*Vibrio* sp. DS40M5, SD004, SD101, SD102, SD248	-	[130]
Amphibactins	*Vibrio* sp. R-10	Amphiphilic cell associated siderophores. This feature can be ascribed to the membrane affinities of amphibactins, which range from 3.8 × 10^3^ to 8.3 × 10^2^ M^−1^, clearly exceeding those for other amphiphilic siderophores.	[131]
Anachelins	*Anabaena cylindrica* NIES-19	-	[122,123]
Anguibactin	*Vibrio anguillarum* 775	Backbone derived from ω-N-hydroxyhistamine, cysteine, and 2,3- dihydroxybenzoic acid. Producing strain is a fish pathogen	[132]
Aquachelins	*Halomonas aquamarina* DS40M3	Contain a given peptidic head group that coordinates Fe(III), alongside with an appendage of a fatty acid moiety. Aquachelins display low critical micelle concentration. Production by open ocean bacteria	[133,134]
Bisucaberin	*Alteromonas haloplanktis*, *Vibrio salmonicida* (fish pathogen)	Anti-tumor activity	[135,136]
Desferrioxamine G	Strain BLI-41	Structurally similar to desferrioxamine B, but for the substitution of a terminal methyl group by a propionic acid moiety	[137]
Fusigen	*Aureobasidium pullulans* HN6.2	Anti-bacterial activity tested against the pathogen *Vibrio anguillarum*	[138]
Loihichelins A–F	*Halomonas* sp. LOB-5	Potential role in the promotion of Mn(II) and Fe(II) oxidation	[139]
Marinobactins	*Marinobacter* sp. DS40M6 and DS40M8	In the presence of Fe(III) marinobactins undergo a spontaneous phase change to form vesicles. In the absence of iron, they are present as micelles at concentration over the critical micelle concentration.	[133]
Ochrobactins A–C	*Ochrobactrum* sp. SP18	Membrane-associated citrate-type photoreactive siderophore, amphiphilic derivatives of aerobactin	[140]
Petrobactin and sulfonated derivatives thereof	*Marinobacter hydrocarbonoclasticus*, *Marinobacter aquaeolei*	Sulfonation takes place in an aromatic ring of petrobactin and is considered to increase the water solubility of the aromatic compounds, as well as to reduce the oxidation of the catechol ring and affect the Fe(III) stability constant	[139,141,142]
Pistillarin	*Penicillium bilaii*	Rare siderophore, one of the two findings of this compound as a natural product. Contains the unusual 3,4-dihydroxycatechol moiety, that is also found in petrobactin	[129]
Pyoverdine	*Pseudomonas aeruginosa* ID 4365	Inhibitory of the growth of fungal plant pathogens	[143]
Rhizoferrin	*Cuninghamella elegans* ATCC36112	Polycarboxylate siderophore	[127]
Schizokinen	*Anabaena* sp. PCC 7120	-	[144]
Synechobactins A–C	*Synechococcus* sp. PCC 7002	Schizokinen derivatives with amphiphilic nature, where an hydroxamic acid is replaced by a long fatty acid, these siderophores are suggested to fix into the membrane of cyanobacteria, given the high affinity, therefore preventing its loss by diffusion into marine environments	[121,131]
Vibrioferrin	*Marinobacter* sp. DG870, 879, 893, and 979	Stoichiometrical boron binding ability through the α-hydroxy- carboxylic acid groups. The photoproduct of this photosensitive siderophore lacks affinity for iron, hence leading to the destruction of the ligand, in contrast with other photosensitive siderophores, where the photoproduct can still coordinate and sequester Fe(III). This characteristic has been suggested to contribute for the mutualistic sharing of iron between marine bacteria and phytoplankton	[107,108]
Vulnibactin	*Vibrio vulnificus* (marine pathogen, causer of causing lethal septicemia or wound infections in humans)	-	[145]

**Table 3:** Typical coordination structures of siderophores (adapted from [112]).

Siderophore	Coordination with Fe(III)
Amphibactins	Through the hydroxamate groups
Aquachelins, marinobactins, ochrobactins, synechobactins	Through the oxygen atoms of each hydroxamate group and both oxygen atoms of the β-hydroxy aspartic acid (in the aquachelins and marinobactins) or of the citric acid (in the ochrobactins and synechobactins)
Alterobactins and pseudoalterobactins	Through the β-hydroxy aspartate moieties and a catecholate group
Petrobactin and sulfonate derivatives	Through the catecholates and the α-hydroxy acid portion of the citrate backbone
